# Treatment of Experimental Autoimmune Encephalomyelitis with an Inhibitor of Phosphodiesterase-8 (PDE8)

**DOI:** 10.3390/cells11040660

**Published:** 2022-02-14

**Authors:** Chaitali P. Basole, Rebecca K. Nguyen, Katie Lamothe, Puja Billis, Mai Fujiwara, Amanda G. Vang, Robert B. Clark, Paul M. Epstein, Stefan Brocke

**Affiliations:** 1 Department of Immunology, UConn Health, Farmington, CT 06030, USA; chaitalipbasole@gmail.com (C.P.B.); rebecca.k.nguyen@gmail.com (R.K.N.); katie.lamothe@comcast.net (K.L.); pujabillis66@gmail.com (P.B.); mai.fujiwara@gmail.com (M.F.); amanda@fiskaaling.fo (A.G.V.); rclark@uchc.edu (R.B.C.); 2 Department of Cell Biology, UConn Health, Farmington, CT 06030, USA; epstein@uchc.edu

**Keywords:** phosphodiesterase (PDE)8, experimental autoimmune encephalomyelitis (EAE), multiple sclerosis (MS)

## Abstract

After decades of development, inhibitors targeting cyclic nucleotide phosphodiesterases (PDEs) expressed in leukocytes have entered clinical practice for the treatment of inflammatory disorders, with three PDE4 inhibitors being in clinical use as therapeutics for psoriasis, psoriatic arthritis, chronic obstructive pulmonary disease and atopic dermatitis. In contrast, the PDE8 family that is upregulated in pro-inflammatory T cells is a largely unexplored therapeutic target. We have previously demonstrated a role for the PDE8A-Raf-1 kinase complex in the regulation of myelin oligodendrocyte glycoprotein peptide 35–55 (MOG_35–55_) activated CD4^+^ effector T cell adhesion and locomotion by a mechanism that differs from PDE4 activity. In this study, we explored the in vivo treatment of experimental autoimmune encephalomyelitis (EAE), a model for multiple sclerosis (MS) induced in mice immunized with MOG using the PDE8-selective inhibitor PF-04957325. For treatment in vivo, mice with EAE were either subcutaneously (s.c.) injected three times daily (10 mg/kg/dose), or were implanted subcutaneously with Alzet mini-osmotic pumps to deliver the PDE8 inhibitor (15.5 mg/kg/day). The mice were scored daily for clinical signs of paresis and paralysis which were characteristic of EAE. We observed the suppression of the clinical signs of EAE and a reduction of inflammatory lesion formation in the CNS by histopathological analysis through the determination of the numbers of mononuclear cells isolated from the spinal cord of mice with EAE. The PDE8 inhibitor treatment reduces the accumulation of both encephalitogenic Th1 and Th17 T cells in the CNS. Our study demonstrates the efficacy of targeting PDE8 as a treatment of autoimmune inflammation in vivo by reducing the inflammatory lesion load.

## 1. Introduction

With more than 800 members characterized in humans, G protein-coupled receptors (GPCRs) constitute the largest class of membrane receptors. Including their downstream signaling pathways, they are the targets for nearly 35% of all approved therapeutics [1]. Approximately two-thirds of the currently druggable GPCRs regulate cAMP signaling, which is a major target of potent anti-inflammatory drugs [1]. cAMP is a regulator of many physiological functions, and is a well-established regulator of immune responses and inflammation [2]. Extracellular ligand binding to Gs-coupled GPCRs leads to cAMP synthesis via the activation of adenylyl cyclase and the conversion of ATP to cAMP. T cell activation leads to a temporary upregulation of cAMP, which is then degraded by cyclic nucleotide phosphodiesterase (PDE) enzymes [3]. PDEs are the only enzymes which are known to hydrolyze cAMP and thus maintain spatial and temporal control over the cAMP gradients within a cell [4]. PDEs are divided into 11 different gene families based on their specificity for cAMP or cGMP, structural similarity, and mode of regulation [5]. While PDEs have been recognized early as potential drug targets for anti-inflammatory drugs, bringing specific PDE inhibitors into clinical use has faced decades of challenges, mostly due to dose-limiting emetic side effects. The approval and clinical use of PDE4 inhibitors for the treatment of major human inflammatory disorders demonstrates the tremendous progress over the last 7 years, with indications expanding at an almost yearly pace. PDE4-selective inhibitors have now been approved for the oral treatment of chronic obstructive pulmonary disease [6], psoriatic arthritis and plaque psoriasis [7], and as a topical treatment for atopic dermatitis [8]; they are also being considered as therapeutics for diseases of the CNS [9,10]. Hence, PDE inhibitors are proving to be of great clinical benefit in inflammatory disorders.

Only a small number of PDEs are currently targeted by approved therapeutics, and there is a significant knowledge gap regarding the specific and potentially therapeutic role of other PDE isoforms. This led to an intense interest in the development of novel PDE inhibitors, including those for the treatment of inflammation. Recently, there have been numerous studies on the cAMP-specific PDE8 family of enzymes encoded by the PDE8A and PDE8B genes, with some studies focusing on the potential of PDE8A as a target for the treatment of inflammation [11,12,13,14,15,16,17,18,19,20,21,22,23,24,25,26]. PDE8A and B are expressed widely in human tissue [27], with functions identified in steroidogenesis [28], lymphocyte adhesion, and chemotaxis [13,16,21,23,29], and a strong association with immune protection against intracellular pathogens [30], brain disorders associated with inflammation [31], and T cells in systemic lupus erythematosus (SLE) [32]. A recent GWAS analysis demonstrated SNPs in the *PDE8* region to be associated with Sjögren’s Syndrome [33]. T cell activation induces PDE8A [24], a cAMP-specific PDE with 40–100-fold greater affinity for cAMP than PDE4 [34]. This unique characteristic has led to the suggestion that PDE8 enzymes may have an important role in the protection of any associated protein from fluctuations in basal cAMP concentrations. Functionally, it has been shown that PDE8A controls T cell and breast cancer cell motility, including adhesion to endothelial cells under physiological shear stress and chemotaxis [13,16,21,23,29]. Additionally, the regulation of T cell adhesion and migration is a selective function of PDE8 that is not shared by PDE4 [14]. 

Myelin oligodendrocyte glycoprotein peptide 35–55 (MOG_35–55_) activated effector T (Teff) cells induce experimental autoimmune encephalomyelitis (EAE), an animal model for multiple sclerosis (MS) [35]. The EAE model recapitulates many features which are characteristic of human MS, including chronic autoimmune inflammation, CNS demyelination and paralysis [36,37]. T cells play a critical role in the induction of EAE, and are thought to be a major driver of MS pathology as well [36,37]. The therapeutic efficacy of anti-migratory drugs targeting a number of different molecules on pathogenic T cells has been demonstrated in vitro and in vivo in EAE and MS [38,39,40]. This EAE model is a standard preclinical animal model for the investigation of pathologic mechanisms of MS, and to test potential drug candidates [36,37]. However, despite their clinical use to treat inflammation in a variety of inflammatory diseases, PDE4 inhibitors have not been approved for the treatment of MS [41]. Previous findings that PDE8A and its isoforms are expressed at higher levels in naive and MOG_35–55_ activated effector T (Teff) cells compared to regulatory T (Treg) cells, and that PDE8 inhibition specifically affects MOG_35–55_ activated Teff cell adhesion, suggest that PDE8 inhibition could be a novel therapeutic approach for the targeting of pathogenic Teff cells in CNS inflammation [13,14]. However, these observations of PDE8 as a target for blocking Teff cell motility were largely based on in vitro and ex vivo experiments [13,14,16,21,23,29]. In order to fill this gap, and to determine whether PDE8 is a potential target for anti-inflammatory therapy in vivo, we tested the inhibition of PDE8 in a MOG_35–55_ induced model of EAE. We explored targeting PDE8 in vivo using the highly selective PDE8 inhibitor PF-04957325 (IC_50_ for PDE8A = 0.0007 μM, PDE8B < 0.0003 μM, other PDE isoforms > 1.5 μM) [21,42]. 

## 2. Materials and Methods

Animals: Female 4–6-week old C57BL/6 mice, the most widely used species and strain for EAE studies [36,37], were obtained from Jackson laboratories (Bar Harbor, ME, USA) and used for the EAE studies. The experiments were performed according to the approved protocols at UConn Health (IACUC Protocol number 100794).

Peptides for EAE induction: The MOG_35−55_ peptide corresponding to the mouse sequence (MEVGWYRSPFSRVVHLYRNGK) was synthesized by the Yale and the Hebrew University Peptide Synthesis Facilities [43]. This peptide is a certified autoantigen for the induction of EAE [35].

Reagents and chemicals used: Complete Freund’s Adjuvant (CFA; Sigma-Aldrich, St. Louis, MO, USA), *Mycobacterium tuberculosis* (Difco Laboratories, Sparks, MD, USA), Collagenase D (Roche Diagnostics, Indianapolis, IN, USA), DNase I (Sigma, St. Louis, MO, USA), Percoll (70%/37%; GE Healthcare, Piscataway, NJ, USA), RBC Lysis Buffer (ThermoFisher Scientific, Agawam, MA, USA), Foxp3/Transcription factor staining buffer set (Affymetrix, Santa Clara, CA, USA), Phorbol 12-myristate 13-acetate (PMA) (Sigma), Ionomycin (Sigma), Brefeldin A (Sigma), BD Cytofix/Cytoperm and BD Perm/Wash buffer (BD Biosciences, San Diego, CA), Paraformaldehyde (Fisher Scientific, Agawam, MA), and Dimethylsulfoxide (DMSO) (Sigma) were the chemicals used. The specific flow cytometry reagents are listed under the respective method sections.

Induction of EAE (active EAE): We used our standard protocol for the induction of EAE [35]. Briefly, six-to-eight-week old female mice were immunized with MOG_35−55_ in Complete Freund’s Adjuvant (CFA; Sigma-Aldrich) as previously described by us [35]. A total of 200 μg MOG_35−55_ peptide and 400 μg killed *Mycobacterium tuberculosis* (Difco Laboratories) was emulsified in CFA and injected subcutaneously (s.c.) into the flanks of the mice. Additionally, each mouse was injected with 200 ng pertussis toxin (PTX; List Biological Laboratories Inc., Campbell, CA, USA) intraperitoneally (i.p.) on day 0 and day 2 post-immunization. PTX is a critical component of the immunization procedure, and is widely used to induce EAE in C57BL/6 mice [35].

Clinical Evaluation of EAE: The mice were scored daily according to a non-linear clinical grading scale from 1 to 5, as follows: 0, no disease; 1, tail weakness; 2, hind-limb paresis/marked slowing of righting; 3, complete paralysis of the hind limbs; 4, severe forelimb weakness/paralysis; 5, moribund or dead [35]. A mean of the daily disease scores (MDD) in each group was calculated over the entire treatment period based on the number of treatment days [40,44].

Treatment: The PDE8-selective inhibitor PF-04957325 was synthesized by Pfizer Inc., Groton, CT [21], and was provided through a Material Transfer Agreement. The inhibitor was dissolved in a stock solution of 50% DMSO and 50% PBS, and was stored in aliquots until use in an experiment. For the long-term treatment in vivo experiments, the mice were either injected subcutaneously three times daily for 6 to 13 days (10 mg/kg/dose), or were implanted subcutaneously with Alzet mini-osmotic pumps filled with PF-04957325 (15.5 mg/kg/day) or a vehicle control (an equivalent volume of DMSO and PBS as the corresponding inhibitor doses) for 14 days. The mice were scored and weighed daily during and after the treatment. For the short-term ex vivo histological analysis of infiltrates, mice with at least a grade 1 disease were treated therapeutically with PF-04957325 (10 mg/kg/dose) or the vehicle control (an equivalent volume of DMSO and PBS to the corresponding inhibitor doses) by subcutaneous injections twice daily for 4 days. For the long-term ex vivo analysis of the infiltrates by flow cytometry, mice with grade 1 or 2 disease were either injected subcutaneously three times daily for 6–13 days (10 mg/kg/dose), or given a vehicle control (an equivalent volume of DMSO and PBS to the corresponding inhibitor doses).

Histology of the brain and spinal cord: The mice were sacrificed by intra-cardiac perfusion with 20 mL cold PBS under isoflurane narcosis. The brain and spinal cord were dissected and fixed by immersion in 10% phosphate-buffered formalin, paraffin embedded, and sectioned (6–7 µM). Three longitudinal sections spanning the entire length of the spinal cord, three cross-sections at different levels in the spinal cord, and four or five sections of brain and brain stem were stained with hematoxylin and eosin (HE) for each animal [35,40]. The total number of inflammatory foci (over 10 mononuclear cells) per visual field in the brain and spinal cord were quantified in the meninges and parenchyma by a double-blinded expert who was not aware of the treatment group of the mice, according to the published protocols [35,45].

Flow cytometry staining: Mice were sacrificed by intra-cardiac perfusion with 20 mL cold PBS under isoflurane narcosis. The spinal cords were dissected and enzymatically digested by incubation with Collagenase D (Roche Diagnostics) and DNase I (Sigma) at 37 °C for 45 min. Mononuclear cells from the spinal cords were isolated by passing them through a cell strainer, followed by Percoll gradient (70%/37%; GE Healthcare) centrifugation [46]. Mononuclear cells derived from lymph nodes and spleens were isolated by passing them through a cell strainer. Red blood cells from spleens were lysed using RBC Lysis Buffer (ThermoFisher Scientific). The total number of mononuclear cells was determined by the trypan blue exclusion method, and was analyzed in a hematocytometer or Vicell counter. For Teff and Treg cell characterization, mononuclear cells were first stained with BD Fc block (clone 2.4G2), followed by staining with cell-surface molecules CD3 (17A2; Affymetrix, Agawam, MA), CD4 (RM4–5; Affymetrix), CD45 (30-F11; Affymetrix) and live–dead blue fluorescent reactive dye (Invitrogen, Carlsbad, CA). The intracellular stainings were performed with transcription factor Foxp3-specific antibodies (FJK-16s; Affymetrix) and Ki-67 (B56; BD Pharmingen) [46] using Foxp3 transcription factor staining buffers (Affymetrix). For the cytokine staining, mononuclear cells were first treated with phorbol 12-myristate 13-acetate (PMA) (Sigma), Ionomycin (Sigma) and brefeldin A (Sigma) for 4 h at 37 °C in order to induce the synthesis of cytokines. The cells were then surface stained, fixed, and permeabilized using Cytofix/Cytoperm and Perm Wash buffer (BD Biosciences). Intracellular staining was carried out with anti-TNF-α (MP6-XT22; eBioscience, San Diego, CA), -IL-17 (TC11-18H10; BD Pharmingen), and -IFN-γ (XMG1.2; Biolegend, San Diego, CA) antibodies, as described [46]. For the staining of the ex vivo restimulated cells with IL-10, the cells were first stained for surface markers and fixed with 4% PFA. The next day, the cells were permeabilized using BD permeabilization buffer, followed by intracellular staining with IL-10 (JES5-16E3; Affymetrix). The percentage of cells, the absolute number of cells and the mean fluorescence intensity (MFI) were determined in a BD LSR II instrument (Flow Cytometry Core, UConn Health) while analyzing the single-cell FACS data using FlowJo software.

Statistical analysis: The treatment effects were assessed by comparing the mean daily disease scores of both experimental groups using the nonparametric Wilcoxon 2 sample analysis/Mann-Whitney *U* test using GraphPad Prism Software. For the comparison of the total number of cells, and the proliferation and cytokine analysis, an unpaired t test was used. Horizontal lines indicate comparisons between samples or periods of analysis: *p* > 0.05 (NS), *p* < 0.05 (*), *p* < 0.01 (**), *p* < 0.001 (***), and *p* < 0.0001 (****).

Data availability: The accession numbers for the data deposited into the public database had not yet been obtained at the time of submission, but will be provided prior to publication.

## 3. Results

### 3.1. PF-04957325 Ameliorates Active EAE in a Therapeutic Fashion

**Figure 1:**
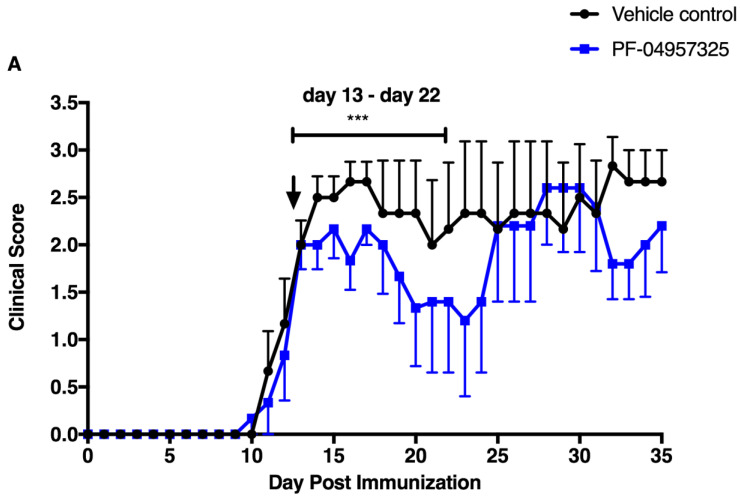

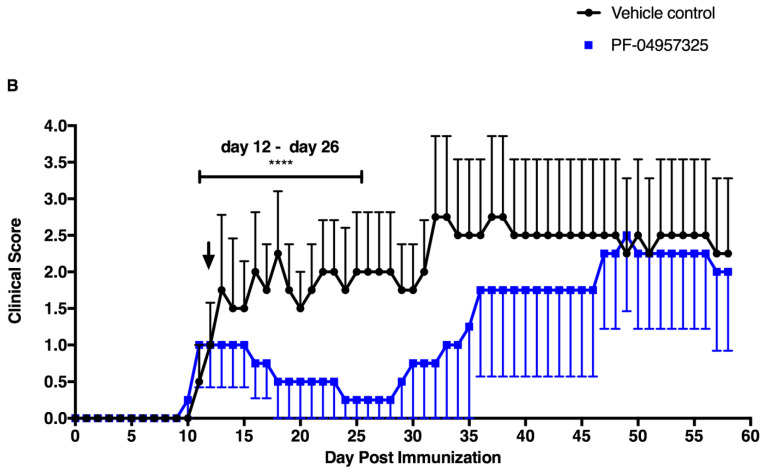
Treatment with the PDE8 inhibitor PF-04957325 treats clinical signs of EAE in a therapeutic manner. (**A**) C57BL/6 mice with an EAE score of at least 1 were treated with the vehicle control in an equivalent volume of DMSO and PBS to the corresponding inhibitor dose (vehicle control), or PF-04957325 (10 mg/kg/dose diluted in DMSO and PBS) subcutaneously 3 times per day, from day 13 to day 22, as indicated by the bar in the figure. The arrow indicates the day on which the treatments were initiated. The data represent the mean clinical scores (mean ± SEM) for each day (*n* = 6 mice per group) *** *p* = 0.0007. (**B**) C57BL/6 EAE mice were implanted with Alzet mini-osmotic pumps filled with either the vehicle alone (50% DMSO and 50% PBS) or PF-04957325 (with a continuous release rate of 15.5 mg/kg/day) in a vehicle on day 12 post-immunization. The treatment duration based on the physical properties of the pumps was 14 days, as indicated by the bar in the figure. The arrow indicates the day on which the treatments were initiated. The data represent the mean clinical scores (mean ± SEM) for each day (*n* = four mice per group) **** *p* < 0.0001.

In order to examine whether the PDE8-mediated regulation of CD4^+^ T cell motility acts on inflammatory disease in vivo, we tested the effect of PF-04957325 in a T cell-mediated MS model, EAE. Active EAE was induced in C57BL/6 mice using MOG_35–55_/CFA and PTX. Once the mice reached a grade 1 or grade 2 of clinical disease, they were treated with a vehicle control or PF-04957325 subcutaneously three times daily for 10 days, from day 13 to day 22 after immunization. We observed a moderate clinical suppression of disease in the PF-04957325-treated group compared to the mice in the vehicle-treated group (Figure 1A). However, the mice in the PF-04957325 group again increased in disease severity once the treatment was stopped. As the half-life of the PDE8 inhibitor is just a few hours, we decided to use a drug delivery mode that provides more stable plasma levels over the treatment period. For continuous drug delivery, we surgically implanted osmotic mini-pumps (Alzet pumps) filled with the vehicle control or PF-04957325 into the subcutaneous cavities of active EAE mice with grade 1 or 2 disease. These pumps are designed to release the drug once they are in the body cavity at a flow rate of 0.5 μL/h for approximately 14 days. In the active EAE model, PF-04957352-treated mice showed the amelioration of their clinical disease during the treatment period (day 12 to day 26) compared to the vehicle-treated mice (Figure 1B). The clinical scores of the mice were monitored daily, and after the 14-day treatment period, the osmotic pumps were removed. As observed earlier, once the treatment was halted, the mice in the PF-04957325-treated group had clinical disease similar to the mice in the vehicle-treated group. 

### 3.2. Treatment with PF-04957325 Suppresses Inflammatory Lesion Formation in the Spinal Cord and Brain, but Not in the Periphery

**Figure 2:**
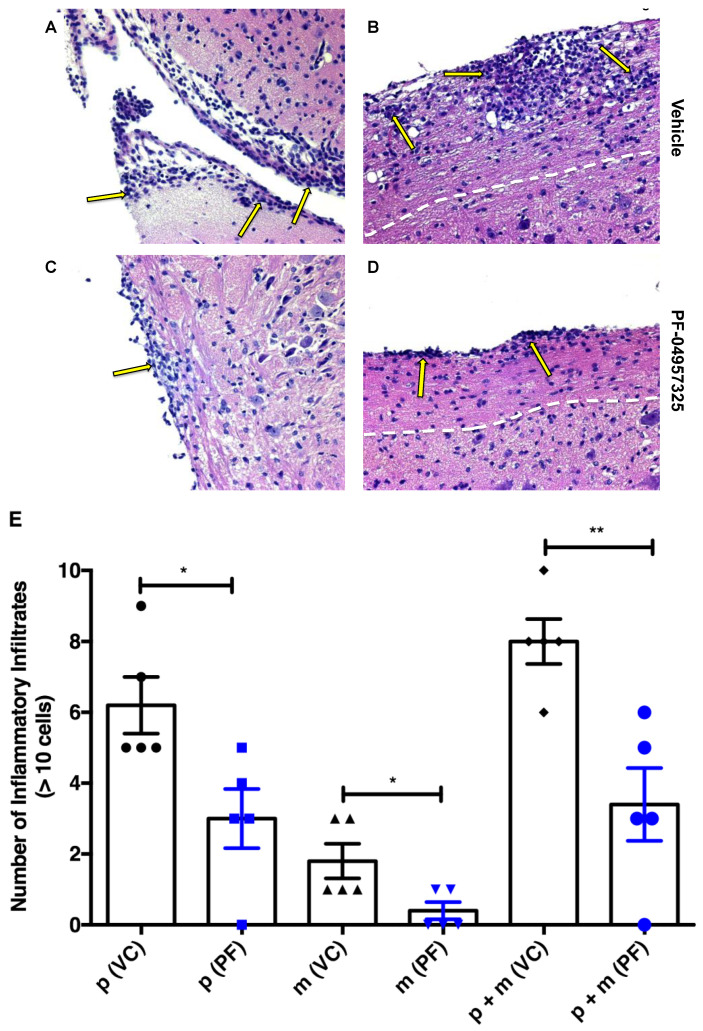
Treatment with PF-04957325 suppresses histopathologic signs of EAE in the CNS. Mice with an EAE score of at least 1 were treated with a vehicle control in an equivalent volume of DMSO and PBS to the corresponding inhibitor dose or PF-04957325 from day 10 to day 13 (twice daily for 4 days), followed by the dissection of their brains and spinal cords for histopathological analysis. The sections were stained with HE, and their inflammatory foci were quantitated according to the published protocols [35,45]. Images of the brain (**A**,**C**) and spinal cord (**B**,**D**) sections of the vehicle control (grade 4) and PF-04957325 (grade 2)-treated mice are shown (original magnification 20X). The arrows point to inflammatory foci, and the white lines indicate the border between white and grey matter in the spinal cord. (**E**) The graph represents the number of inflammatory foci containing >10 cells [35,45] present in the spinal cord parenchyma (p) (* *p* = 0.025), brain meninges (m) (* *p* = 0.034), and spinal cord parenchyma plus brain meninges (p + m) (** *p* = 0.005), as assessed in HE sections of the vehicle control (V)- and PF-04957325 (PF)-treated mice. (*n* = five mice per group).

In order to test whether the inhibition of PDE8 in vivo reduced the accumulation of cells in the CNS during EAE, we treated mice with an EAE score of at least 1 twice daily with the vehicle control or PF-04957325 for 4 days. The HE staining of the brain and spinal cord sections on day 4 of treatment were performed in order to obtain a quantitative assessment of the infiltrates in the CNS (Figure 2A–E). There were significant reductions in the number of infiltrates in the brain meninges and spinal cord parenchyma, as well as in the combined meningeal and parenchymal lesion load after treatment with PF-04957325 (Figure 2E). 

**Figure 3:**
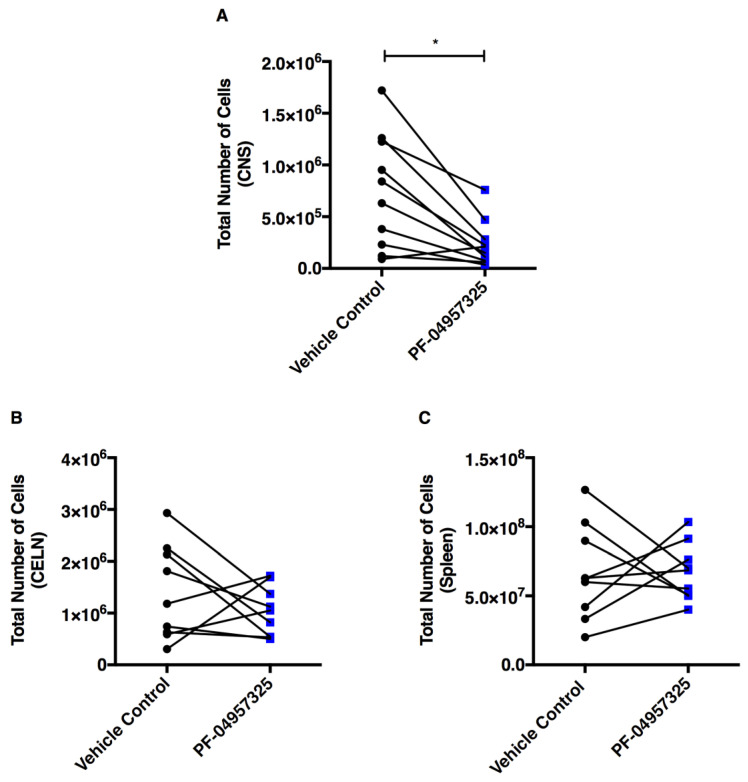
Treatment with PF-04957325 suppresses leukocyte infiltration into the spinal cord. Mice with EAE scores of at least 1 were treated with the vehicle control or PF-04957325 three times daily, for 6 to 13 days. The data represent the total number of mononuclear cells in the spinal cord (CNS) after Percoll gradient isolation (**A**) (*n* =10 mice per group, five independent experiments; * *p* = 0.015)), in cervical lymph nodes (CELN) (**B**), and spleens (**C**) (*n* = 9 mice per group, mean ± SEM of four independent experiments) counted by trypan blue exclusion assay. The horizontal lines indicate comparisons between each pair of vehicle control- or PF-04957325-treated mice enrolled in the treatment regimen at the same time.

We also determined the total number of live mononuclear cells isolated from the spinal cords of mice with EAE treated with PF-04957325 using the trypan exclusion blue assay. The mice were treated for 6 to 13 days, which led to a significant reduction in the total amount of mononuclear cell accumulation in the spinal cord (7.45 × 10^5^ vs. 2.38 × 10^5^) (Figure 3A). The treatments did not significantly affect the total number of cells accumulating in the cervical lymph nodes (CELN) (1.39 × 10^6^ vs. 1.03 × 10^6^) or in the spleen (66.68 × 10^6^ vs. 67.28 × 10^6^) (Figure 3B,C).

### 3.3. Treatment with PF-04957325 Suppresses CD4^+^ Teff Cell and Treg Cell Accumulation in the Spinal Cord

**Figure 4:**
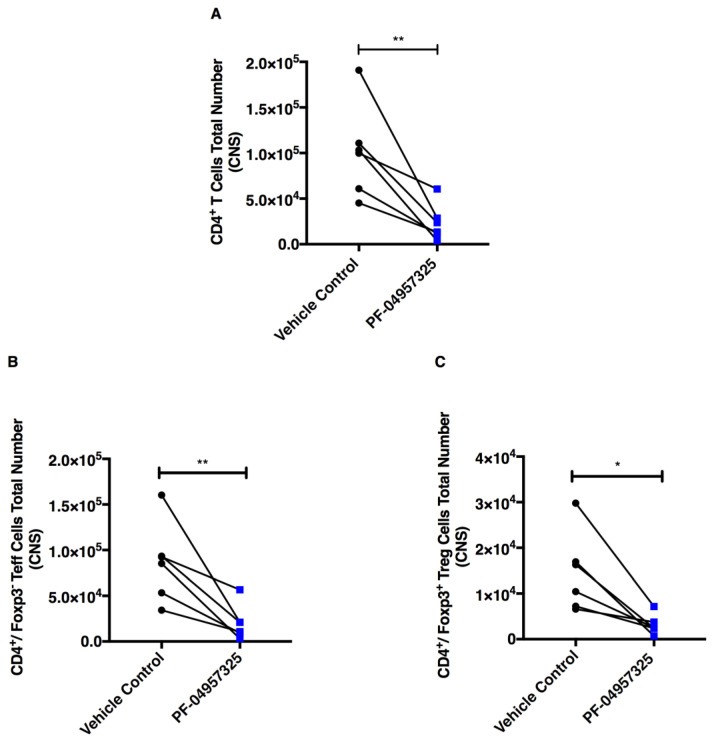
Treatment with PF-04957325 suppresses the accumulation of total CD4^+^ T cells and Teff and Treg cell subpopulations in the spinal cord. Mice with EAE scores of at least 1 were treated with the vehicle control or PF-04957325 thrice daily for 6 to 13 days. The data represent the total number of CD4^+^ T cells (** *p* = 0.006) (**A**), Foxp3^−^/Teff cells (** *p* = 0.007) (**B**), and Foxp3^+^/Treg cells (* *p* = 0.01) (**C**) in the spinal cord (CNS) analyzed by flow cytometry (*n* = six mice per group, mean ± SEM of four independent experiments).

In order to assess whether the accumulation of specific CD4^+^ T cells in the spinal cord was affected by the treatment, we performed a detailed FACS analysis of the mononuclear cells isolated from the spinal cord of EAE mice after treatment with PF-04957325 (Figure S1). PDE8 inhibition in vivo does not change the percentage of CD4^+^ T cells, CD4^+^/Foxp3^−^ Teff cells and CD4^+^/Foxp3^+^ Treg cells accumulating in the spinal cord after 6 to 13 days of treatment (data not shown). We observed a significant decrease of the total number of CD4^+^ T cells (1.02 × 10^5^ vs. 2.37 × 10^4^), CD4^+^/Foxp3^−^ Teff cells (8.65 × 10^4^ vs. 2.04 × 10^4^) and CD4^+^/Foxp3^+^ Treg cells (1.46 × 10^4^ vs. 3.17 × 10^3^) (Figure 4A–C). 

### 3.4. Treatment with PF-04957325 Suppresses the Accumulation of Activated and Proliferating Teff and Treg Cell Subpopulations in the Spinal Cord

**Figure 5:**
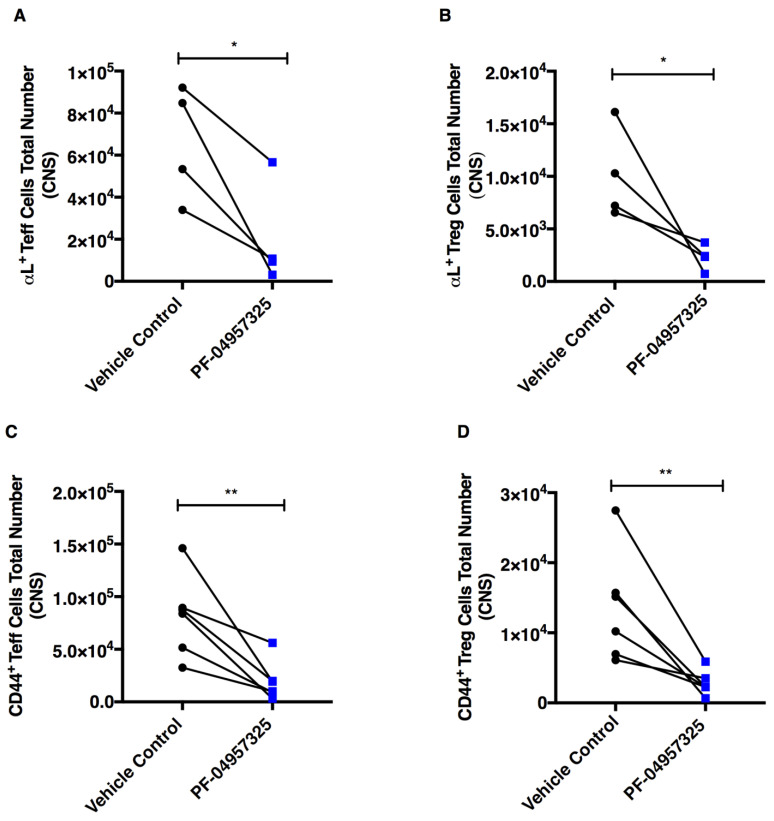

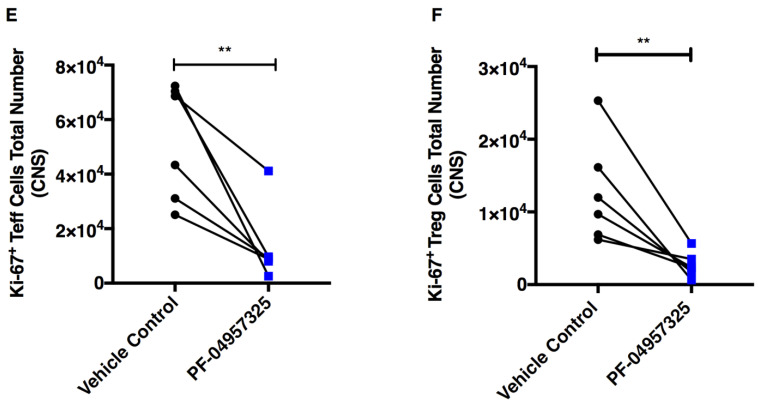
Treatment with PF-04957325 suppresses the accumulation of activated and proliferating Teff and Treg cell subpopulations in the spinal cord. Mice with EAE scores of at least 1 were treated with the vehicle control or PF-04957325 thrice daily for 6 to 13 days. The data represent the total number of Foxp3^−^/αL ^+^ Teff cells (* *p* = 0.046) (**A**), Foxp3^+^/αL^+^ Treg cells (* *p* = 0.014) (**B**) (*n* = four mice per group, mean ± SEM of four independent experiments), Foxp3^−^/CD44^+^ Teff cells (** *p* = 0.006) (**C**), Foxp3^+^/CD44^+^ Treg cells (** *p* = 0.008) (**D**), Foxp3^−^/Ki-67^+^ Teff cells (** *p* = 0.0040) (**E**) and Foxp3^+^/Ki-67^+^ Treg cells (** *p* = 0.008) (**F**) (*n* = 6 mice per group, mean ± SEM of four independent experiments) in the spinal cord (CNS), analyzed by flow cytometry.

In order to assess whether PDE8 inhibition in vivo was affecting the expression of activation markers and adhesion molecules [40] on T cells within the spinal cord, we analyzed the percentage and total number of αL integrin^+^, CD44^+^ and Ki-67^+^ cells within the Teff and Treg cell populations (Figure 5). We observed a significant decrease in total number of αL integrin^+^ Teff cells (6.61 × 10^4^ vs. 2 × 10^4^) and αL integrin^+^ Treg cells (1 × 10^4^ vs. 2.3 × 10^3^) (Figure 5A,B), CD44^+^ Teff cells (8.18 × 10^4^ vs. 1.96 × 10^4^) and CD44^+^ Treg cells (1.36 × 10^4^ vs. 2.81 × 10^3^) (Figure 5C,D). We also saw a significant decrease in the total number of Ki-67^+^ Teff cells (5.18 × 10^4^ vs. 1.33 × 10^4^) and Ki-67^+^ Treg cells (1.27 × 10^4^ vs. 2.71 × 10^3^) (Figure 5E,F) in the spinal cord after treatment with PF-04957325.

### 3.5. Treatment with PF-04957325 Suppresses the Accumulation of Pro-Inflammatory T Cell Subsets in the Spinal Cord

**Figure 6:**
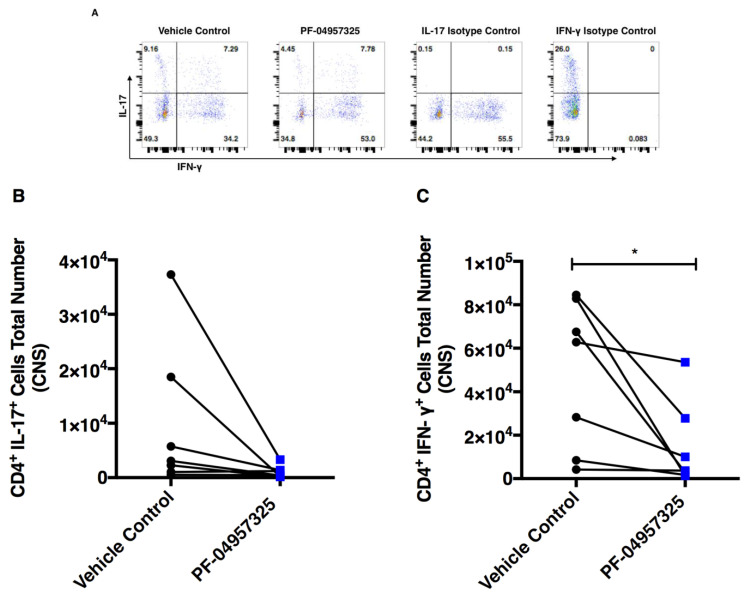
Treatment with PF-04957325 suppresses the accumulation of pro-inflammatory Th1 cells in the spinal cord. Mice with EAE scores of at least 1 were treated with the vehicle control or PF-04957325 thrice daily, for 6 to 13 days. (**A**) The detection of IL-17^+^ and IFN-γ^+^ CD4^+^ cells after ex vivo restimulation with PMA/Ionomycin by flow cytometry in the spinal cord mononuclear cells of EAE mice treated with the vehicle control or PF-04957325. The representative dot plots show CD4^+^ T cells that are positive for IL-17 (*y*-axis) and IFN-γ^+^ (*x*-axis). The data represent the total number of IL-17^+^ (**B**) and IFN-γ^+^ CD4^+^ cells (* *p* = 0.04) (**C**) in the spinal cord (CNS). (**B**,**C**) *n* = 7 mice per group; mean ± SEM of four independent experiments.

In order to assess the effect of PDE8 inhibition on inflammation in the spinal cord and periphery, we analyzed the pro-inflammatory and anti-inflammatory cytokine synthesis of T cell subsets ex vivo after PMA/ionomycin stimulation. Representative dot plots of CD4^+^ T cells that are positive for IL-17 (*y*-axis) and IFN-γ^+^ (*x*-axis) are shown in Figure 6A. We found that 6 to 13 days of treatment with PF-04957325 lead to a statistically non-significant decrease in the total number of IL-17^+^ CD4^+^ T cells (0.97 × 10^4^ vs. 0.1 × 10^4^) (Figure 6B), and a significant decrease in IFN-γ^+^ CD4^+^ T cells (4.84 × 10^4^ vs. 1.44 × 10^4^) in the spinal cord (Figure 6C). 

**Figure 7:**
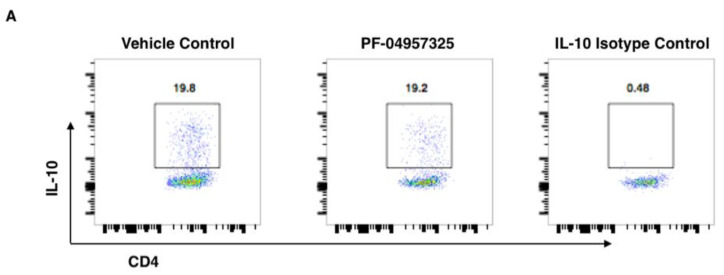

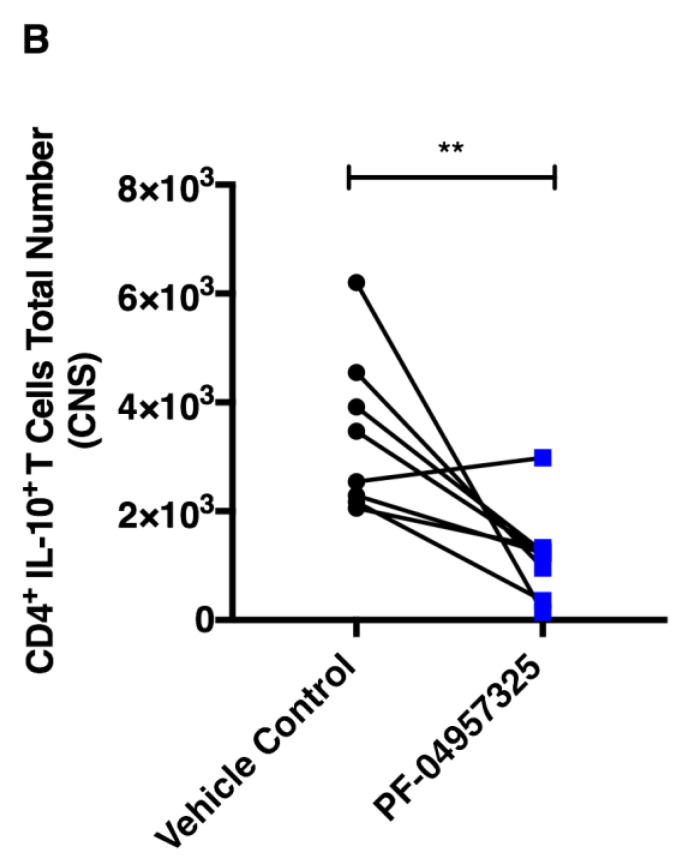
Treatment with PF-04957325 suppresses the accumulation of IL-10-producing CD4^+^ T cells in the spinal cord. Mice with EAE scores of at least 1 were treated with the vehicle control or PF-04957325 thrice daily, for 6 to 13 days. (**A**) The detection of IL-10^+^ CD4^+^ cells after ex vivo restimulation with PMA/Ionomycin by flow cytometry in the spinal cord mononuclear cells (CNS) of EAE mice treated with the vehicle control or PF-04957325. The representative dot plots show that CD4^+^ T cells (*x*-axis) that are positive for IL-10^+^ (*y*-axis) show comparable frequencies in the vehicle- and inhibitor-treated mice (** *p* = 0.002). (**B**) The data represent the total number of IL-10^+^ CD4^+^ T cells (*n* = eight mice per group, mean ± SEM of three independent experiments).

Because we observed a clinical suppression of disease after long-term treatment, we tested whether there were any changes in IL-10 production in CD4^+^ T cells (Figure 7). There was a significant decrease in the total number (3.4 × 10^3^ vs. 1.19 × 10^3^) of IL-10-producing CD4^+^ T cells (Figure 7B), but no decrease in the proportion of IL-10-producing cells (Figure 7A). 

### 3.6. Treatment with PF-04957325 Does Not Affect Pro-Inflammatory Cytokine Production in the Spleen and CELN

In order to assess the effect of PDE8 inhibition on the peripheral immune system, we analyzed the pro-inflammatory cytokine production of T cell subsets in the CELN and spleen after PMA/ionomycin stimulation. We found that 6 to 13 days of treatment with PF-04957325 does not significantly affect the total number of IL-17^+^, IFN-γ^+^, and TNF-α^+^ CD4^+^ T cells in the CELN (Figure S2A–C) or the spleen (Figure S3A–C). Additionally, the total number of IL-10^+^ CD4^+^ T cells in the spleen and CELN were not significantly changed during the treatment (Figure S4A,B).

## 4. Discussion

Early studies on the characterization of PDE in human lymphocytes led to the concept that they could serve as excellent targets for anti-inflammatory therapy, focusing on PDE4 as the predominantly expressed isoform [47]. Since this early work, PDE inhibitors have been investigated for the treatment of autoimmune disorders [48]. However, bringing PDE inhibitors to treat inflammation into the clinics has faced decades of challenges, mostly due to the emetic side effects of otherwise-effective PDE4 inhibitors [41,49]. The initial clinical trials with PDE4 inhibitors were unsuccessful, including those in MS patients [41,50]. The knowledge gap of the specific actions of each of the PDE isoforms in immune cells [48,51] provides a strong rationale for the evaluation of selective inhibitors targeting the different PDE gene families, in addition to *PDE4,* in order to treat diseases of the immune system [51].

Our study evaluated PDE8-directed treatment for in vivo inflammation for the first time, and indicated that it is a potent target to suppress inflammation in the CNS in a therapeutic manner. The EAE model was chosen as a standard pre-clinical model to test potential new MS therapies [36,37,52]. Treatment with PF-04957325 reduced the clinical signs of EAE. We observed a significant reduction of the clinical disease scores across the acute chronic phase of the disease. The treatment effects recorded in our experiments were reversible, as suppression largely correlated with the period of drug administration in vivo. This could be due to the short half-life of the inhibitor in vivo, or its characteristics as a reversible-type inhibitor [42]. Most PDE inhibitors are reversible, binding to the enzyme through non-covalent bonds and acting on the cAMP binding site of the catalytic moiety. Under these conditions, the affected cells can recover their enzymatic activity quickly [53]. These common features of small molecule inhibitors of PDEs allow for the relatively precise dosing and control of the duration of the treatment. While these characteristics underlie the need to take PDE inhibitors daily, they could in turn provide benefits with respect to the side effects of immunosuppressive therapies, such as natalizumab in patients who are at risk for progressive multifocal leukoencephalopathy (PML) due to impaired immune surveillance [54]. Because of their short on and off rates and oral availability, PDE inhibitors can be dosed very precisely, and can be quickly removed from the circulation should such side effects occur [49].

In an effort to define the mechanisms by which PF-04957325 ameliorates EAE, we found that treatment with PF-04957325 reduced the histopathologic signs of EAE by reducing inflammatory lesion formation in the brain and spinal cord of mice with EAE. In EAE and relpasing-remitting MS, the lesion load is closely correlated with disease severity [36,37,41]. In our experiments, the number of inflammatory foci was reduced in the areas that are most affected in EAE pathology, i.e. the leptomeninges and the white matter of the CNS. These results indicate that the accumulation of disease-mediating leukocyte populations is profoundly affected by treatment with PF-04957325. Additionally, the analysis of the specific pathogenic leukocyte populations demonstrated that the number of CD4+ Teff cells, as well as the number of Th1 T cells, was reduced within the spinal cords of mice treated with the PDE8 inhibitor. These findings were accompanied by the reduction of T cells carrying markers which are characteristic of activated T cells associated with disease pathology. Here, we attempted to elucidate the mechanism by which PDE8 inhibition reduces cell migration into the CNS. PDE8 inhibition may block CD4^+^ T cell infiltration into the spinal cord, possibly by affecting the integrin- or CD44-mediated adhesion of CD4^+^ Teff cells to the vascular endothelial cells of the blood–brain barrier. Indeed, the number of integrin and CD44-positive T cells in the spinal cord is reduced after treatment with PF-04957325. Once these CD4^+^ T cells enter the spinal cord, their reactivation might also be blocked by PF-04957325. We have shown previously that the disruption of the PDE8-Raf-1 complex suppresses Teff cell adhesion by affecting LFA-1 integrin-mediated cell tethers with ICAM-1. Additionally, because the LFA-ICAM-1 axis is a crucial part of T cell activation at the immunological synapse, it is possible that, in vivo, the CD4^+^ Teff interaction with antigen-presenting cells is affected. This might explain the reduction in the total number of Th1 cells and proliferating CD4^+^ Teff cells observed in the spinal cord. The inhibitor has a selective effect in the CNS, as we did not see a significant effect on cell numbers or cytokine production in the periphery after 6 to 13 days of treatment. 

The failure of clinical trials to treat primary progressive MS with the PDE4 inhibitor rolipram suggests that other PDE families, such as PDE8, expressed in Teff cells might be important for controlling their pro-inflammatory function. Our previous work has shown that PDE8 is important for chemotaxis of T cells and their adhesion to endothelial cells [13,21,23]. PDE8 has a very high affinity for cAMP with a km value in the range of 40–150 nM (40 fold higher than that of PDE4), and hence might function at lower cAMP concentrations than PDE4 [26]. PDE8A is insensitive to inhibition by the non-specific PDE inhibitor IBMX but is inhibited by the PDE inhibitor dipyridimole (DP) (IC_50_ in the range of 4–9 μM) [5]. Work from our lab has shown that treatment with dipyridamole inhibits the chemotaxis of activated splenocytes [23], whereas the inhibition of PDE3, PDE4 or PDE7 does not affect the chemoattractant-induced migration of these activated cells. Because dipyridamole also inhibits PDE8, this was the first evidence that PDE8 might be an important regulator of migration. Supporting a role for PDE8 in controlling EAE, dipyridamole treatment at high doses (100–300 mg/kg) leads to a reduction in EAE severity and microglial reactivity in the CNS [55]. 

PDE8A is expressed in activated CD4^+^ T cells, and we have shown that it regulates the adhesion of MOG_35–55_ activated CD4^+^ effector T cells to inflamed brain endothelial cells under physiological flow conditions [13]. PDE8A forms a complex with Raf-1 kinase and protects it from cAMP effector protein PKA-mediated phosphorylation [19]. We have previously shown that the disruption of the PDE8A-Raf-1 kinase complex also inhibits the migration of MOG_35–55_ activated CD4^+^ effector T cells to inflamed brain endothelium under shear flow conditions [13]. PDE4 inhibitors increased the adhesion of CD4^+^ T cells, and when added along with PDE8 inhibitor reduced the suppression of adhesion [14]. This suggests that PDE8 and PDE4 regulate different pools of cAMP in CD4^+^ T cells, and hence regulate different cAMP-mediated functions. Our previous studies have demonstrated that PDE8 does not affect T cells’ proliferation or ex vivo cytokine production but specifically targets the motility of CD4^+^ T cells [21]. We have also shown that PDE8 is expressed at higher levels in the acute inflammatory stage compared to the tolerant stage in lung draining hilar lymph nodes in an ovalbumin-induced allergic airway disease model [14]. Collectively, our current observations and previous evidence point towards a unique therapeutic mechanism that urges further study targeting PDE8 in inflammatory diseases. 

The selective effects of PDE8 inhibition on Teff cells’ infiltration in the spinal cord could be a beneficial stand-alone option to treat CNS autoimmunity. Additionally, the combination treatment of a PDE4 with a PDE8 inhibitor may afford the use of lower doses of the PDE4 inhibitor, and may thereby mitigate limitations due to its considerable side effects [41,49]. Hoffman and colleagues recently discovered a dual PDE4/8 inhibitor, BC8-15 (the IC_50_ for PDE8A and PDE4 is 0.28 μM and 0.22 μM, respectively) [28]. Of note, ibudilast, a weak inhibitor of multiple PDE isoforms, is currently being tested for efficacy in relapsing-remitting and progressive MS [56,57]. The effects of combined PDE inhibitors on the clinical suppression of disease and their mechanism of action need to be established in future studies. In conclusion, our present study demonstrates the efficacy of targeting PDE8 as a treatment of autoimmune inflammation in vivo by reducing the inflammatory lesion load in the CNS and the clinical signs of EAE.

## Data Availability

The data will be deposited in a publicly accessible database, and a link will be provided during the review.

## Supplementary Materials

The following supporting information can be downloaded at: https://www.mdpi.com/article/10.3390/cells11040660/s1, Figure S1: Gating strategy for flow cytometry analysis of T cells isolated from the spinal cord of mice with EAE; Figure S2: Treatment with the PF-04957325 does not affect pro-inflammatory cytokine production in the cervical lymph nodes; Figure S3: Treatment with the PF-04957325 does not affect pro-inflammatory cytokine production in the spleen; Figure S4: Treatment with the PF-04957325 does not affect anti-inflammatory cytokine production in the cervical lymph nodes and spleen.
