# Supplementary material for: Treatment of Experimental Autoimmune Encephalomyelitis with an Inhibitor of Phosphodiesterase-8 (PDE8)
Source: Cells. 2022 Feb 14;11(4):660. doi: 10.3390/cells11040660 (PMC8870644; doi:10.3390/cells11040660)
Supplement: Supplementary file 1 [file cells-11-00660-s001.zip › cells-1562335-SI.pdf]

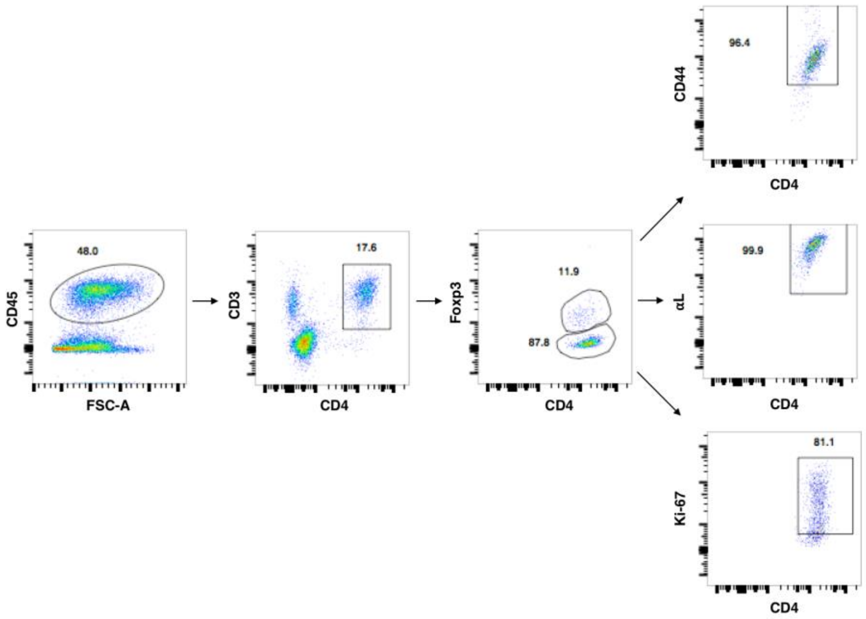

Figure S1. Gating strategy for flow cytometry analysis of T cells isolated from the spinal cord of mice with EAE. Representative flow cytometry plots showing the gating strategy for detection of Foxp3$^-$ Teff cells, Foxp3$^+$ Treg cells, αL$^+$, CD44$^+$ and Ki-67$^+$ Teff and Treg cells in spinal cord mononuclear cells (CNS) of EAE mice treated with vehicle control or PF-04957325.

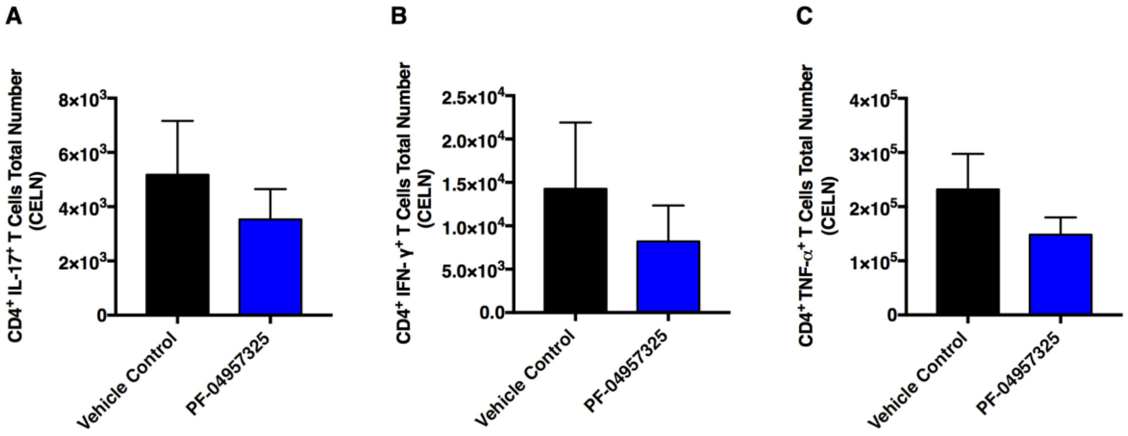

Figure S2. Treatment with the PF-04957325 does not affect pro-inflammatory cytokine production in the cervical lymph nodes.

Data represents the total number of IL-17$^+$ (A), IFN-γ$^+$ (B), and TNF-α$^+$ CD4$^+$ T cells (C) in the cervical lymph nodes (CELN) after *ex vivo* restimulation with PMA/Ionomycin. (n = 6 mice per group, mean ± SEM of 3 independent experiments).

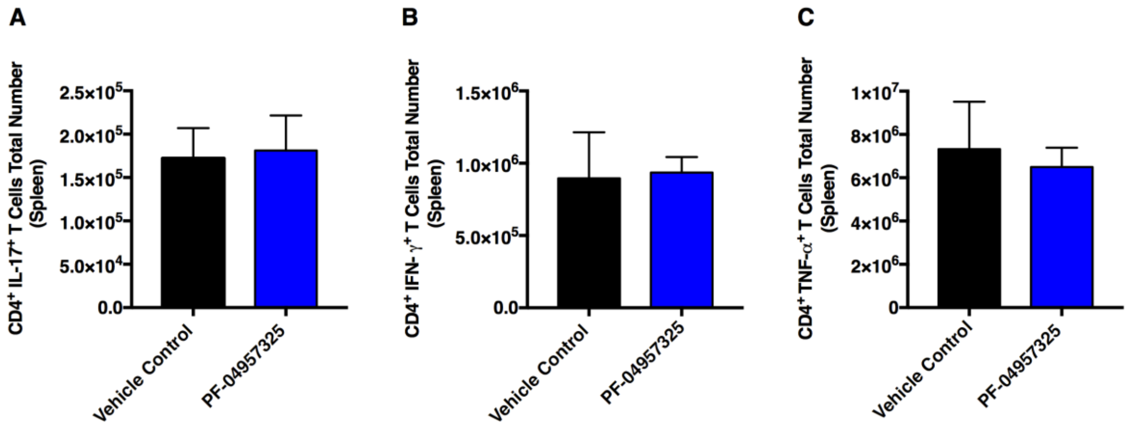

Figure S3. Treatment with the PF-04957325 does not affect pro-inflammatory cytokine production in the spleen.
Data represents the total number of IL-17^+ (A), IFN-γ^+ (B), and TNF-α^+ (C) $CD4^+$ T cells in the spleen after *ex vivo* restimulation with PMA/Ionomycin. (n = 5 mice per group, mean ± SEM of 2 independent experiments).

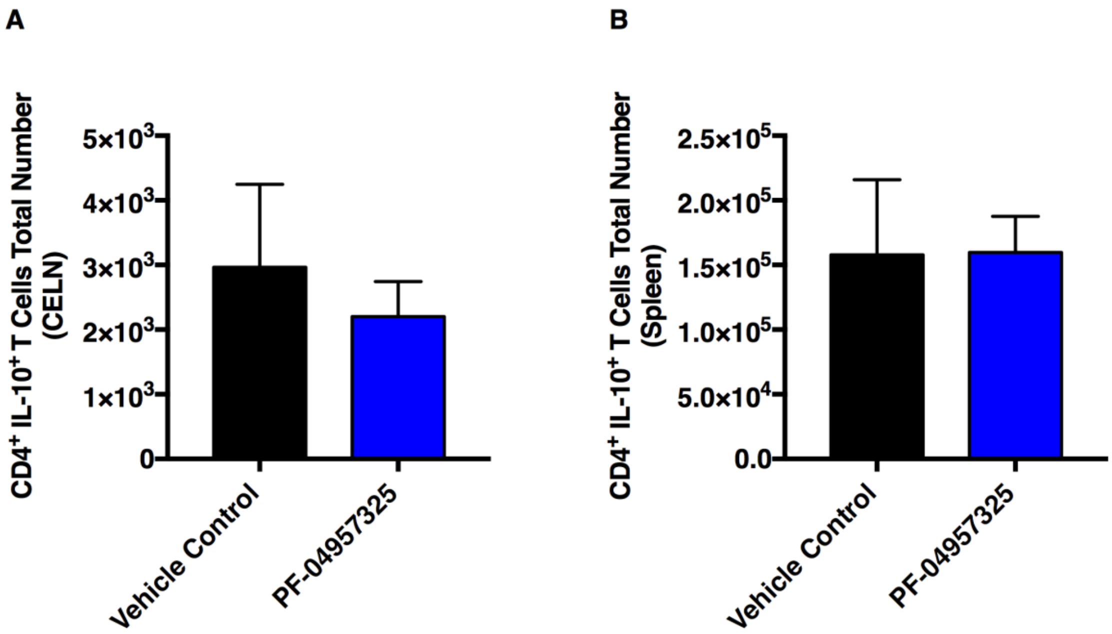

Figure S4. Treatment with the PF-04957325 does not affect anti-inflammatory cytokine production in the cervical lymph nodes and spleen.
Data represents the total number of IL-10^+ $CD4^+$ T cells in the cervical lymph nodes (CELN) (A) and spleen (B) after *ex vivo* restimulation with PMA/Ionomycin. (n = 7 - 8 mice per group, mean ± SEM of 3 independent experiments)
